# Regenerative Potential of Various Plant‐Derived Exosome Injections in Laser‐Induced Skin Wound Healing in a Rabbit Model

**DOI:** 10.1111/jocd.70561

**Published:** 2025-11-24

**Authors:** Noury Adel, Jack Kolenda, Jesper Thulesen, Nenad Stankovic, Francisco Llano, Ida Vega Thulesen, Anna Jo, Martin Braun, Alyaa Ragaei Hassan

**Affiliations:** ^1^ Oral and Maxillofacial Surgery Specialist Private Practice Cairo Egypt; ^2^ Department of Otolaryngology Head and Neck Surgery University of Toronto Toronto Ontario Canada; ^3^ Private Practice Copenhagen Denmark; ^4^ Private Practice Belgrade Serbia; ^5^ Private Practice Mexico City Mexico; ^6^ Eye Department Roskilde University Hospital Roskilde Denmark; ^7^ Orsayun Clinic Seoul South Korea; ^8^ Private Practice Vancouver British Columbia Canada; ^9^ Saxony Egypt University for Applied Sciences and Technology Cairo Egypt

**Keywords:** angiogenesis, CD31, collagen, exosomes, laser‐induced skin wound, plant‐derived exosomes, wound healing

## Abstract

**Background:**

Thermal injuries caused by laser incisions in soft tissue can delay wound healing by triggering inflammation and tissue damage. Plant‐derived exosomes have recently gained attention as innovative biological agents with promising regenerative capabilities for soft tissue repair.

**Objective:**

This study investigates the effectiveness of injecting three commercial plant‐based exosome products: Exoline, Glow, and Elysee in enhancing the healing process of laser‐induced skin wounds in a rabbit model.

**Methods:**

One hundred and twenty adult male New Zealand rabbits were randomized into four groups (*n* = 30 each): untreated control, Exoline, Glow, and Elysee. Full‐thickness 1 × 1 cm laser wounds were created on the ventral aspect of the right ear. Exosome‐treated groups received 0.1 mL injections into the wound bed and margins immediately after injury. Animals were sacrificed at baseline (Day 0) to confirm comparable wound conditions across groups, and subsequently at Day 7 and Day 14. Wound healing was assessed grossly and histologically using hematoxylin and eosin, Masson's trichrome (for collagen fiber deposition), and CD31 immunohistochemistry (for angiogenesis). Quantitative scoring was performed by blinded observers.

**Results:**

Clinically, all wounds achieved apparent closure by Day 14; however, histological assessment revealed persistent differences in tissue organization and angiogenesis between groups. At Day 7, Glow induced the highest collagen deposition, followed by Elysee and Exoline, whereas Exoline elicited the strongest angiogenic response, followed by Elysee and Glow. By Day 14, Glow maintained the lead in collagen maturation, followed by Exoline and Elysee, while Exoline remained most effective for angiogenesis, followed by Elysee and Glow. All exosome‐treated groups significantly outperformed controls (*p* < 0.05).

**Conclusion:**

Plant‐derived exosomes enhance cutaneous wound repair with formulation‐specific benefits. Glow optimized early and late collagen deposition, Exoline promoted angiogenesis, and Elysee contributed to intermediate angiogenic effects. These findings support the potential for tailored exosome‐based therapies in regenerative dermatology. These preliminary results in a rabbit model warrant further investigation in long‐term studies and human clinical applications.

## 1 Introduction

The integration of laser technology into surgical procedures for soft tissue manipulation has revolutionized clinical practice by offering a precise, minimally invasive approach that reduces intraoperative bleeding and enhances operator control. Despite these clear advantages, the application of laser energy inevitably induces localized thermal trauma to adjacent tissues. This thermal insult results in structural and cellular alterations that can complicate and potentially delay the natural wound healing process. Understanding the biological impact of laser‐induced injury and identifying effective strategies to support tissue repair remain critical challenges in both experimental and clinical contexts [1, 2, 3].

Animal models play a vital role in unraveling the complex biology of skin wound healing. Among these, the rabbit ear model stands out due to its anatomical and physiological similarities to human skin, including comparable epidermal thickness and rich vascularization. This model allows for standardized wound induction and controlled assessment of therapeutic interventions. Moreover, the thin dermal layer and limited subcutaneous fat provide an excellent platform for evaluating regenerative approaches and scar formation in a reproducible manner. The utilization of laser incisions in this model provides a clinically relevant approximation of the thermal injuries often encountered in surgical settings, enabling detailed investigation of healing dynamics under controlled experimental conditions [4, 5, 6, 7].

Thermal injury from laser application initiates a cascade of cellular damage primarily through protein denaturation, disruption of cell membranes, and microvascular injury. These changes precipitate an acute inflammatory response characterized by infiltration of neutrophils and macrophages, the release of pro‐inflammatory cytokines such as interleukin‐1 beta (IL‐1β) and tumor necrosis factor‐alpha (TNF‐α), and an increase in oxidative stress. While inflammation is a necessary phase of wound healing, excessive or prolonged inflammatory activity can exacerbate tissue destruction, impair re‐epithelialization, and delay the transition to subsequent proliferative and remodeling phases. The restoration of skin integrity depends on a finely tuned balance between these biological processes, involving the coordinated deposition of extracellular matrix components like collagen, fibroblast activation, and angiogenesis [8, 9, 10].

In recent years, the role of extracellular vesicles, particularly exosomes, in mediating tissue repair has attracted considerable interest. Exosomes are nanosized vesicles secreted by cells that carry a diverse cargo of proteins, lipids, nucleic acids, and microRNAs capable of modulating recipient cell behavior. Mammalian cell‐derived exosomes, especially those from mesenchymal stem cells, have demonstrated significant potential in enhancing wound repair by promoting angiogenesis, reducing inflammation, and stimulating proliferation of skin cells. However, their clinical application is limited by challenges including complex isolation procedures, concerns about immunogenicity, ethical considerations related to stem cell sources, and high manufacturing costs [11, 12, 13, 14, 15].

Plant‐derived exosomes emerge as a promising alternative therapeutic platform due to their natural abundance, ease of scalable production, and intrinsic bioactive properties. These vesicles, isolated from various plant sources, encapsulate a variety of beneficial compounds such as antioxidants, flavonoids, and small RNAs, which can influence mammalian cellular pathways involved in inflammation resolution, extracellular matrix remodeling, and neovascularization. Importantly, plant exosomes have demonstrated excellent biocompatibility with minimal risk of adverse immune reactions, positioning them as attractive candidates for regenerative medicine applications, including topical or localized treatments for skin injuries [16, 17, 18, 19, 20].

Despite growing evidence supporting the regenerative capabilities of plant‐based exosomes, there remains a scarcity of comparative data evaluating the effectiveness of different commercial formulations in models of laser‐induced skin damage. Such comparative studies are essential to identify the most efficacious products and optimize treatment protocols for clinical translation. This study aims to systematically assess the wound healing potential of three commercially available plant‐derived exosome preparations: Exoline, Glow, and Elysee using a well‐established rabbit ear model of laser‐induced skin wounds. This model offers the advantages of reproducibility, ease of monitoring wound progression, and relevance to human skin physiology.

We hypothesize that administration of plant‐derived exosomes will significantly improve healing outcomes relative to untreated controls by accelerating epithelial regeneration, enhancing collagen deposition, and promoting new blood vessel formation. Furthermore, we anticipate differences in therapeutic efficacy among the tested formulations, reflecting variability in their bioactive cargo composition and delivery mechanisms. By elucidating these comparative effects, our study seeks to advance the understanding of plant exosome‐based interventions and pave the way for their incorporation into future clinical wound management strategies.

## 2 Materials and Methods

### 2.1 Animal Model

The present experimental study was conducted on 120 healthy adult male New Zealand White rabbits ( *Oryctolagus cuniculus* ), chosen due to their well‐characterized skin physiology and common use as a reliable model in cutaneous wound healing research. The rabbits were aged between 5 and 6 months and weighed approximately 2.5 to 3.0 kg. This age range and weight were selected to ensure homogeneity in physiological parameters and to minimize variability related to age or size.

All experimental procedures complied with the ARRIVE (Animal Research: Reporting of In Vivo Experiments) guidelines (version 2.0) to promote ethical and transparent reporting [21]. The study protocol was approved by the Institutional board, ensuring that all animal handling, surgical procedures, and euthanasia methods adhered to internationally recognized ethical standards for the care and use of laboratory animals. The animals were handled by experienced personnel trained in veterinary care and animal welfare to minimize pain, distress, and discomfort throughout the study duration.

### 2.2 Animal Housing and Diet

Upon arrival, the rabbits were acclimatized for 2 weeks under standard laboratory conditions to reduce stress and allow physiological stabilization prior to experimental interventions. They were individually housed in stainless steel cages measuring 60 × 40 × 50 cm, which allowed free movement while preventing aggressive interactions.

Environmental conditions were strictly controlled: temperature was maintained at 21°C–23°C, relative humidity ranged between 55% and 60%, and a 12‐h light/dark cycle was imposed to simulate natural circadian rhythms. The rabbits received a commercial pelleted diet formulated for growing rabbits, ensuring sufficient macro‐ and micronutrients necessary for wound healing processes. Water was provided ad libitum via automated watering systems, ensuring continuous hydration.

The cages were cleaned daily using a non‐toxic, phosphate‐free disinfectant solution to maintain hygiene and prevent secondary infections. Bedding was changed every 48 h. Handling was minimized to essential interventions and conducted by the same trained staff to reduce variability in stress‐induced physiological responses [22].

### 2.3 Sample Size Calculation

Sample size was calculated using G*Power 3.1.9.7. Assuming a moderate effect size (*f* = 0.35), a significance level of 0.05, and a power of 80%, a minimum of 8 animals per group per timepoint was required. To enhance robustness and ensure standardized numbers across intervals, the sample size was increased to 10 animals per group per timepoint.

Thus, the total cohort consisted of 120 rabbits, evenly distributed across four experimental groups and three timepoints (baseline, Day 7, Day 14). This design ensured adequate power for inter‐ and intra‐group comparisons.

This sample size was chosen to ensure adequate statistical power and biological reliability for histological and immunohistochemical comparisons among groups. It also allowed for the detection of meaningful differences in collagen deposition and angiogenesis while accounting for potential sample loss or technical variability during tissue processing. Including 30 rabbits per group (10 per timepoint) enabled within‐ and between‐group comparisons across healing intervals (baseline, Day 7, and Day 14), ensuring consistency and robust temporal analysis of wound healing progression.

### 2.4 Study Design and Animal Allocation

To ensure unbiased distribution and to minimize selection bias. animals were randomized into four equal groups (*n* = 30 per group) using block randomization, at each time point (Day 0, Day 7, Day 14), 10 rabbits from each group were sacrificed for tissue harvesting. At baseline (Day 0), 10 rabbits per group were euthanized to confirm comparable wound conditions prior to exosome administration.

### 2.5 Grouping and Experimental Design

This investigation was structured to include four distinct experimental groups:

- Group 1: Control group (laser‐induced thermal injury without treatment): In this foundational group, rabbits were subjected to a standardized thermal skin injury induced by a 450 nm diode laser applied to the ventral side of the ear. No therapeutic interventions were administered following the laser incision. This group functioned as a natural healing baseline, enabling detailed observation of the intrinsic wound repair process over time. The data collected from this control cohort provided critical insight into the physiological inflammatory response, cellular infiltration, and re‐epithelialization dynamics in the absence of any exogenous treatments.
- Group 2: Exoline exosome treatment group: Following identical laser injury procedures, rabbits in this group received injections of 0.1 mL of Exoline plant‐derived exosomes (manufactured by ABiomaterials, South Korea) at the wound bed and along the wound edges.
- Group 3: Glow exosome treatment group: This group was similarly subjected to the standardized laser injury. Post‐injury, rabbits were treated with 0.1 mL of Glow injections (produced by Louis Derma, France) at the wound bed and along the wound edges.
- Group 4: Elysee exosome treatment group: Rabbits in this group underwent the same laser‐induced skin injury and subsequently received 0.1 mL of Elysee plant‐derived exosome injections (Dermafirm, South Korea) at the wound bed and along the wound edges.

### 2.6 Induction of Standardized Skin Injury

Prior to wounding, rabbits were anesthetized via intramuscular injection of ketamine hydrochloride (35 mg/kg) combined with xylazine (5 mg/kg), following standard anesthesia protocols based on previous research [23]. The ventral ear skin was shaved and sterilized with 70% ethanol to maintain aseptic conditions. Using a 450 nm diode laser (Woodpecker LX16 Plus, Woodpecker Medical Technology Co. Ltd., China), a full‐thickness, 1 × 1 cm wound with an approximate depth of 2 mm was created on the ventral aspect of the right ear of each animal. The wound induction procedure followed previously described methods for laser‐induced skin injury in rabbits [24]. Laser parameters were standardized at a continuous output power of 2 W, with all wounds performed by a single experienced operator to minimize variability. Post‐procedurally, animals did not receive antibiotics or analgesics to avoid confounding the inflammatory and healing response.

### 2.7 Administration of Exosome Treatment

Immediately following laser injury, animals assigned to Groups 2, 3, and 4 received local injections of their respective exosome formulations. Under aseptic conditions, a 31‐gauge insulin syringe was used to inject 0.1 mL of the exosome suspension directly into the wound bed and surrounding peri‐wound tissues, ensuring uniform delivery across the affected area. The control group (Group 1) did not receive any injection, allowing assessment of natural healing processes.

### 2.8 Exosome Preparations

The study utilized three commercial plant‐derived exosome formulations: Exoline (ABiomaterials, South Korea), Glow (Louis Derma, France), and Elysee (Dermafirm, South Korea). According to the manufacturers, each formulation is standardized during production to ensure consistency in particle concentration and bioactivity across batches. However, proprietary restrictions limit the disclosure of detailed characterization data such as nanoparticle size distribution, protein concentration, and RNA cargo. As such, no independent validation of these parameters was performed in the present study. This limitation is acknowledged and highlights the need for future molecular and proteomic analyses to better define the therapeutic properties of these preparations.

### 2.9 Gross Examination of the Wounds

At the end of the experiment (Day 14), wounds from all groups were visually inspected to assess wound closure, presence of scabs, erythema, and signs of infection. This gross examination allowed a preliminary evaluation of wound healing prior to histological processing.

### 2.10 Tissue Harvesting and Sample Processing

At each predefined time point (Days 0, 7, and 14), 10 rabbits from each group were euthanized by intravenous administration of pentobarbital sodium (200 mg/kg) under deep anesthesia, following established guidelines for laboratory animals [25]. The entire wounded area, along with a 2 mm margin of adjacent healthy skin, was carefully excised. Specimens were immediately fixed in 10% neutral buffered formalin for 48 h to preserve cellular morphology and tissue architecture, and then processed using standard histological procedures, including dehydration through graded ethanol series, clearing in xylene, and embedding in paraffin wax, as described in a previous research [26]. Thin tissue sections of 5 μm thickness were cut using a microtome and mounted on glass slides for downstream analysis.

### 2.11 Histological and Immunohistochemical Analysis

#### 2.11.1 Masson's Trichrome Staining

To evaluate collagen deposition and the extent of fibrosis, sections were stained using the Masson's trichrome protocol following standard histological procedures [27]. Collagen fibers were visualized in blue, muscle fibers in red, and cell nuclei in black. Images from five randomly selected high‐power fields (100× magnification) per sample were captured with a light microscope equipped with a digital camera. Quantitative analysis was performed using ImageJ software, calculating the percentage area occupied by collagen relative to total tissue area [28].

#### 2.11.2 CD31 Immunohistochemistry

To assess angiogenesis, immunohistochemical staining for CD31, a widely used endothelial cell marker, was performed following standard protocols [27]. After antigen retrieval and blocking of endogenous peroxidase activity, sections were incubated overnight at 4°C with primary anti‐CD31 antibody (Abcam, Cat# ab28364, Cambridge, UK; dilution 1:100). Detection was achieved using a biotinylated secondary antibody and visualization with 3,3′‐diaminobenzidine (DAB), producing a brown reaction product in endothelial cells.

CD31 expression was evaluated semi‐quantitatively in five randomly selected high‐power fields (400× magnification) per section using an intensity‐based scoring system [29]: 0 = no staining, 1 = weak staining, 2 = moderate staining, 3 = strong staining. This scoring method was selected over optical density or area percentage because it allows for rapid assessment of staining intensity in heterogeneous tissue areas, particularly when vascular structures are discrete and irregular. Weak, moderate, and strong reactions were differentiated based on the visibility and density of stained endothelial cells relative to background tissue: weak = sparse cells with light staining, moderate = multiple cells with moderate brown intensity, strong = dense clusters of cells with intense brown staining. All scoring was performed by two independent blinded observers, and discrepancies were resolved by consensus.

### 2.12 Statistical Analysis

Data were analyzed using IBM SPSS Statistics version 27.0 (IBM Corp., Armonk, NY, USA). Normality of distribution was assessed using the Shapiro–Wilk test, and homogeneity of variances was verified with Levene's test. A two‐way analysis of variance (ANOVA) was conducted to evaluate the effects of treatment group and time on each outcome variable, followed by Tukey's post hoc tests for pairwise comparisons. In addition to reporting means and standard deviations (SD), 95% confidence intervals (CIs) were calculated for all group means. Effect sizes were determined using Cohen's d for pairwise comparisons and partial eta‐squared (*η* ^2^) for ANOVA models, in order to quantify the magnitude of observed effects beyond *p*‐values. Statistical significance was defined as *p* < 0.05.

## 3 Results

The study demonstrated distinct temporal and group‐specific variations in collagen deposition and angiogenesis, as assessed by Masson's trichrome staining and CD31 immunohistochemistry, respectively. Clinical examination revealed that by day 14, all groups including the untreated control showed complete wound closure. However, histological and immunohistochemical analyses highlighted significant differences in extracellular matrix remodeling and vascularization between treatment groups.

### 3.1 Gross Examination of Wounds (Naked Eye Inspection)

At Day 0, wounds in all groups were similar, with no visible differences in size or depth. By Day 7, partial wound closure and granulation tissue formation were observed across all groups, with treated groups showing more pronounced healing than controls. At Day 14, all groups achieved complete clinical closure, although the quality of wound healing varied among treatments.

Examination of wounds at Day 0 revealed comparable baseline morphology across all groups, with no significant differences in collagen deposition or CD31 expression.

### 3.2 Histological and Immunohistochemical Results

#### 3.2.1 Baseline (Day 0)

At Day 0, all groups were comparable. H&E staining showed open wound margins with no epithelialization or granulation tissue. Masson's trichrome staining revealed minimal collagen fibers, thin and loosely organized, while CD31 immunohistochemistry demonstrated negligible endothelial expression. There were no statistically significant differences among groups in collagen deposition or angiogenesis.

#### 3.2.2 Day 7

**FIGURE 1:**
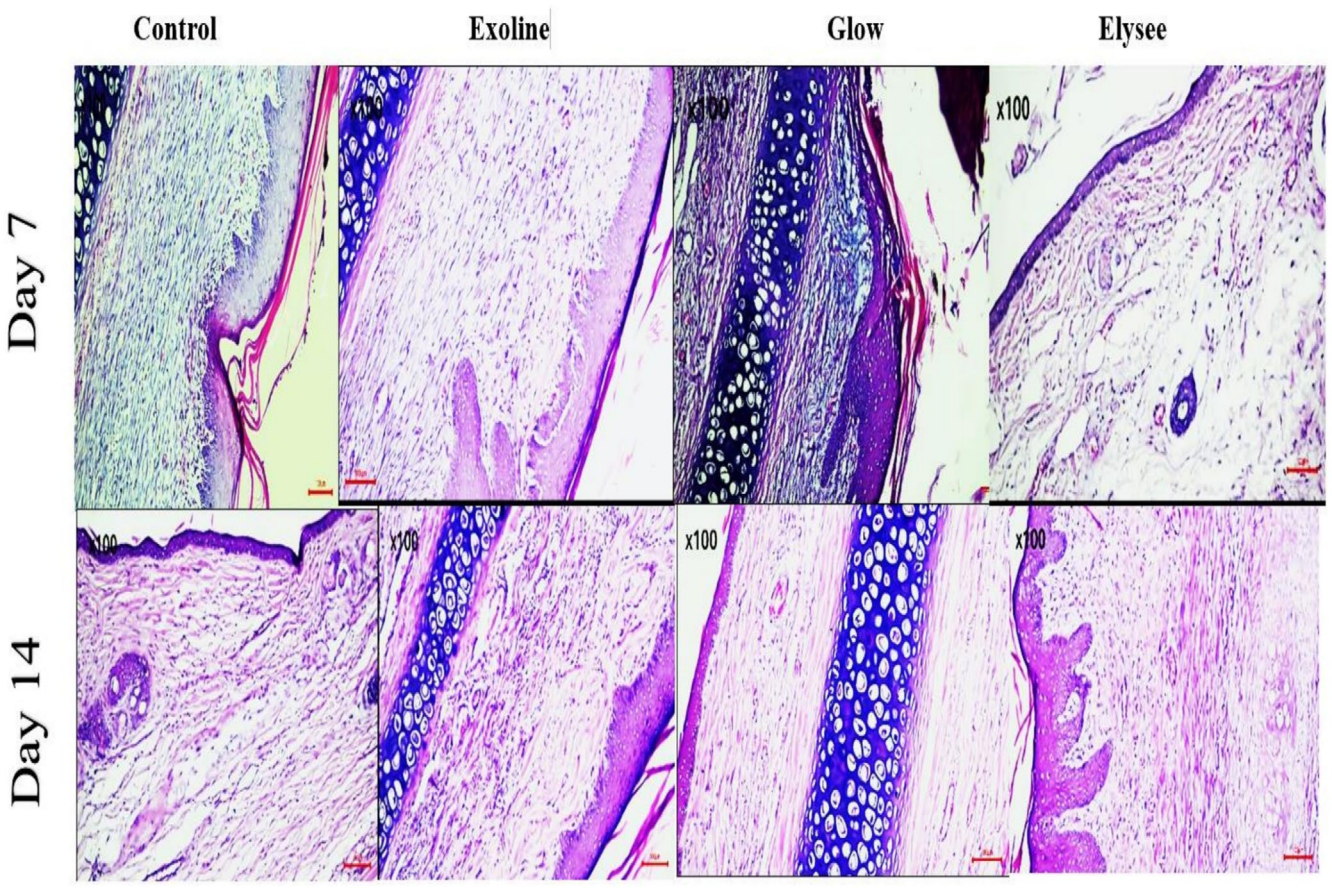
Hematoxylin and eosin (H&E) staining of wounds in all groups at Day 7 and Day 14. Arrows indicate wound margins (scale bar = 100 μm).

**FIGURE 2:**
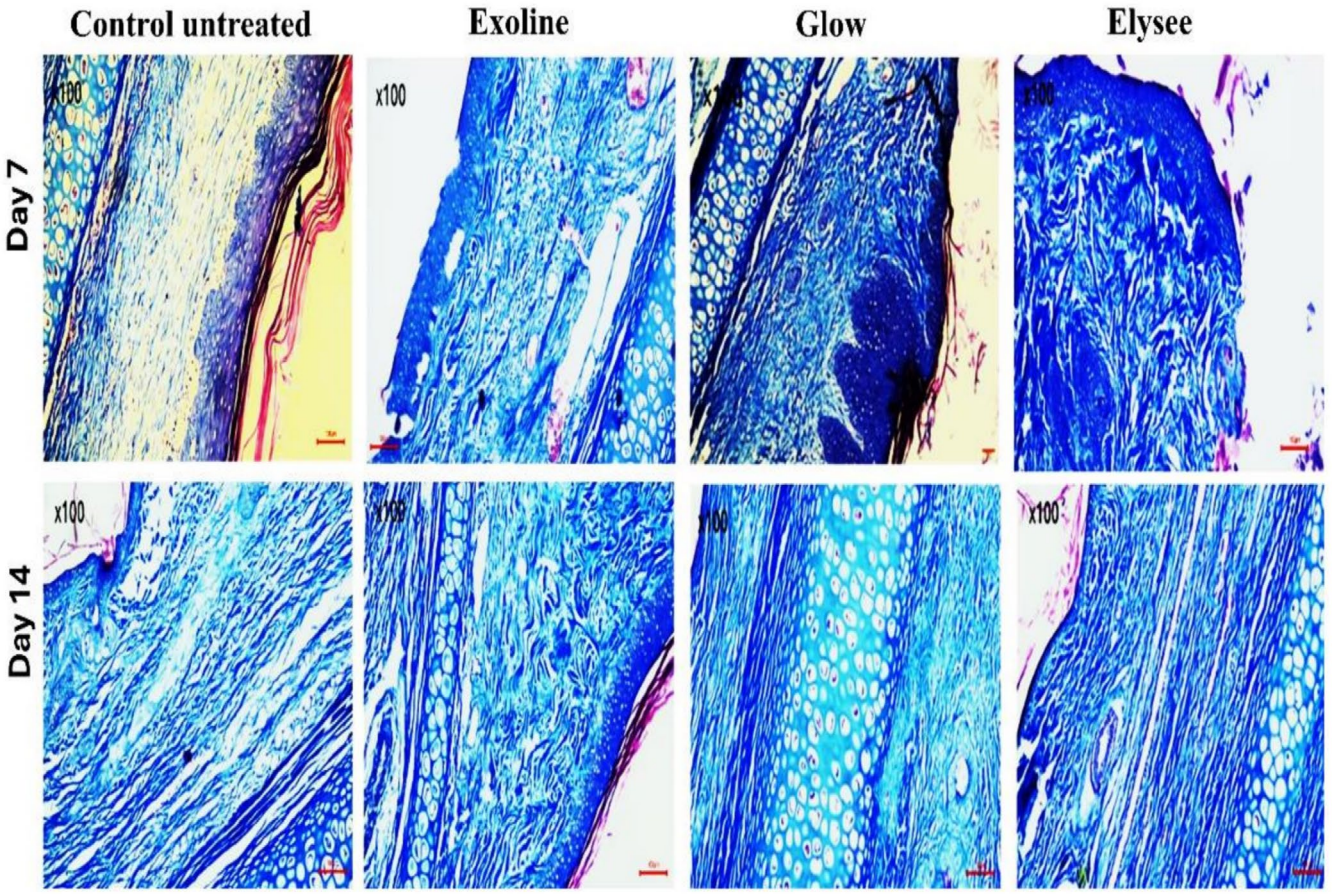
Masson's trichrome (MT) staining of wounds in all groups at Day 7 and Day 14 (scale bar = 100 μm).

**FIGURE 3:**
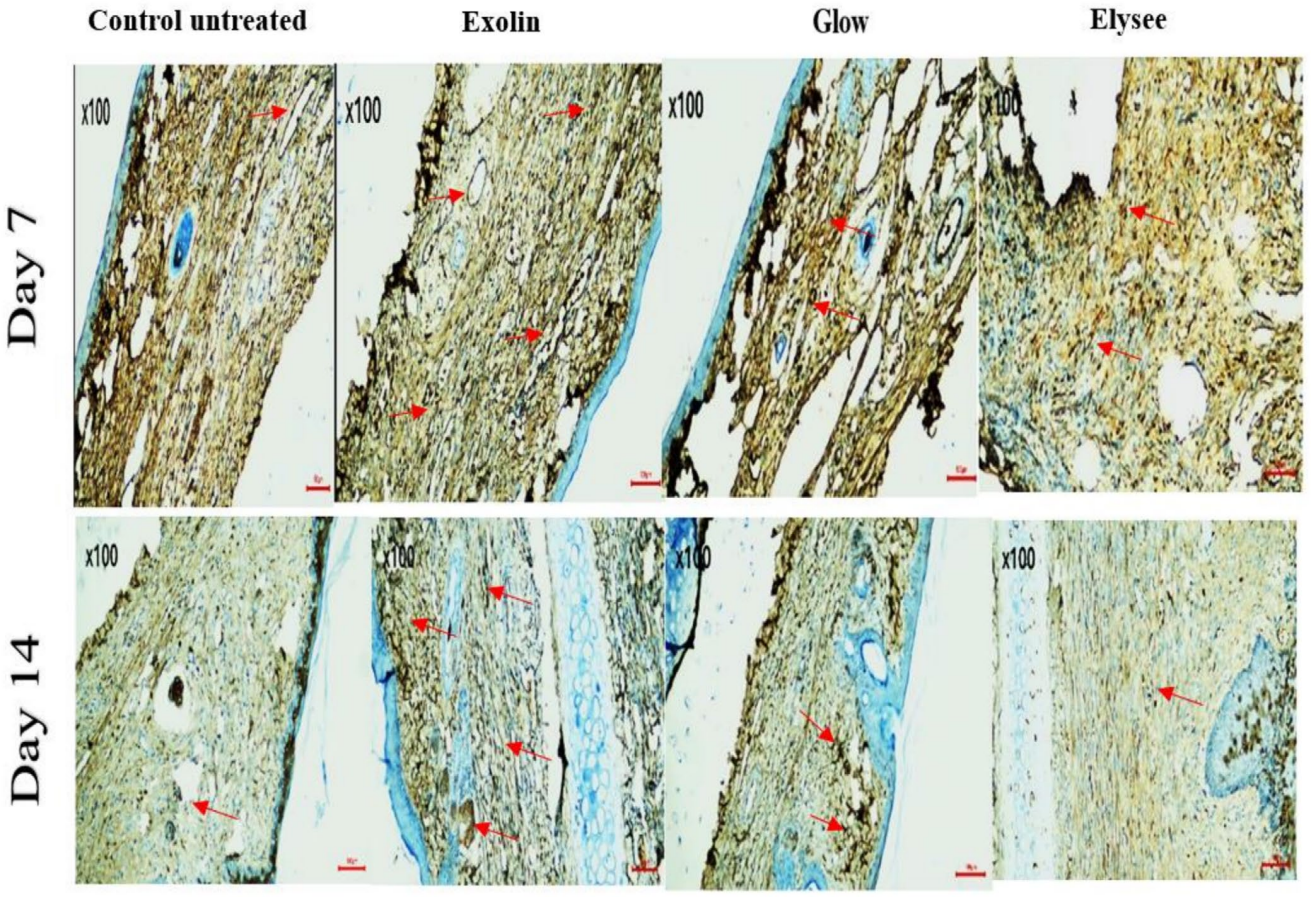
CD31 immunohistochemical staining in all groups at Day 7 and Day 14. Arrows indicate CD31‐positive microvessels (scale bar = 50 μm).

H&E staining at Day 7 indicated partial wound closure in all groups. Glow‐treated wounds had the most advanced granulation tissue with dense fibroblast infiltration, followed by Elysee and Exoline. Control wounds displayed delayed epithelialization with sparse granulation tissue. Masson's trichrome staining revealed that Glow had the highest collagen deposition, followed by Elysee and Exoline, whereas control remained minimal (Figures 1 and 2). CD31 immunostaining showed that Exoline induced the strongest angiogenic response, followed by Elysee and Glow, while control exhibited weak and scattered endothelial labeling (Figure 3).

#### 3.2.3 Day 14

By Day 14, H&E staining confirmed complete wound closure in all groups. Glow‐treated wounds showed the most advanced dermal remodeling, with thick, well‐aligned collagen bundles bridging the wound bed. Elysee and Exoline exhibited intermediate collagen organization, while control wounds had thin, loosely arranged fibers. Masson's trichrome analysis corroborated these findings, with Glow achieving the highest collagen deposition, followed by Exoline and Elysee (Figures 1 and 2). CD31 staining demonstrated that Exoline maintained the strongest angiogenic response, followed by Elysee and Glow, whereas control remained poorly vascularized (Figure 3). These results highlight formulation‐specific effects on tissue regeneration despite similar clinical closure.

### 3.3 Blinded Analysis and Inter Observer Reliability

**FIGURE 4:**
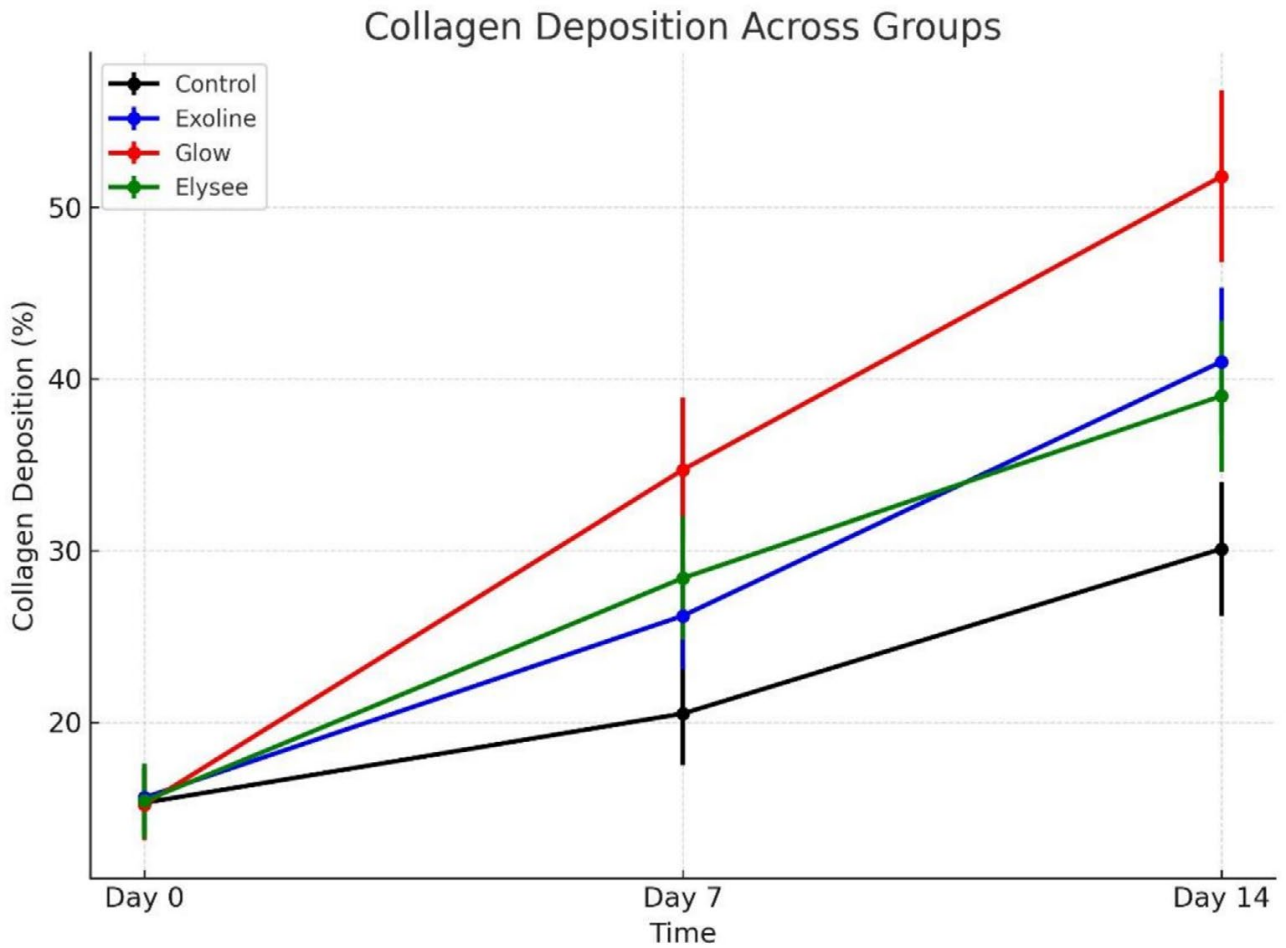
Mean collagen fiber deposition (percentage ± SD) across groups at baseline, Day 7, and Day 14, as assessed by MT staining. No intergroup differences at baseline. By Day 7, Glow‐treated wounds showed highest collagen deposition, followed by Elysee and Exoline (*p* < 0.05). Day 14: Glow maintained lead, followed by Exoline and Elysee (*p* < 0.01).

**FIGURE 5:**
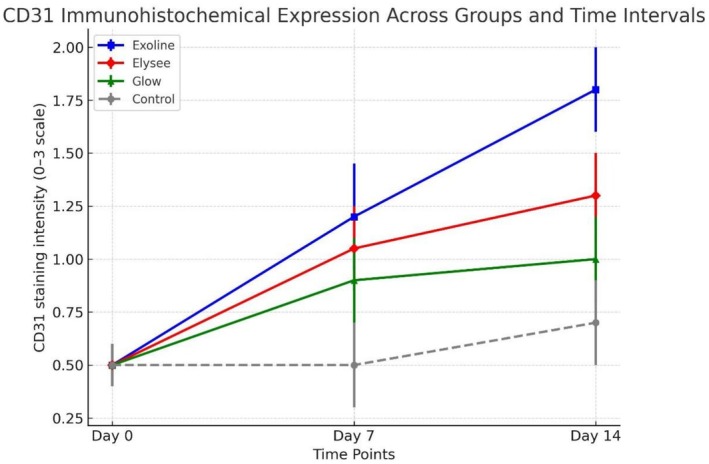
CD31 immunohistochemical expression (intensity score 0–3) across groups at baseline, Day 7, and Day 14. No intergroup differences at baseline. Day 7: Exoline strongest, followed by Elysee and Glow (*p* < 0.05). Day 14: Exoline remains most effective for angiogenesis, followed by Elysee and Glow (*p* < 0.01).

To maintain methodological rigor and eliminate observer bias, all histological and immunohistochemical assessments were independently conducted by two senior histopathologists blinded to group assignment. Quantitative analysis focused on collagen fiber deposition and organization (Masson's trichrome) and CD31 immunohistochemical intensity for angiogenesis. Interobserver reliability was high, with Cohen's kappa coefficients of *κ* = 0.93 for collagen scoring and *κ* = 0.95 for CD31 evaluation, confirming excellent reproducibility and internal data reliability. These findings reinforce the statistical and biological validity of the observed therapeutic effects (Tables 1 and 2; Figures 4 and 5).

**TABLE 1:** Percentage of collagen fiber deposition (mean ± SD; 95% CI) in laser‐induced cutaneous wounds across experimental groups at baseline, Day 7, and Day 14.

Timepoint	Control (*n* = 10)	Exoline (*n* = 10)	Glow (*n* = 10)	Elysee (*n* = 10)	ANOVA *p*‐value	Post hoc comparisons (Cohen's *d*)
Day 0	15.3 ± 2.1 (95% CI: 13.8–16.8)	15.6 ± 2.0 (14.2–17.0)	15.2 ± 2.1 (13.7–16.7)	15.4 ± 2.2 (13.8–17.0)	0.88 (ns)	No significant differences
Day 7	20.5 ± 3.0 (18.3–22.7)	26.2 ± 3.1 (23.9–28.5)	34.7 ± 4.2 (31.6–37.8)	28.4 ± 3.6 (25.9–30.9)	< 0.001	Glow > Elysee (*d* = 1.6) > Exoline (*d* = −0.65) > Control (Glow vs. Control *d* = −3.9)
Day 14	30.1 ± 3.9 (27.2–33.0)	41.0 ± 4.3 (37.8–44.2)	51.8 ± 5.0 (48.1–55.5)	39.0 ± 4.4 (35.7–42.3)	< 0.001	Glow > Exoline (*d* = −2.3) > Elysee (Glow vs. Control *d* = −4.8) > Control

*Note:* One‐way ANOVA with Tukey's post hoc test performed; Cohen's *d* values indicate effect sizes. Glow exosome induced highest collagen deposition at Days 7 and 14; Exoline and Elysee had intermediate effects. Cohen's *d* represents standardized effect size (0.2 = small, 0.5 = medium, 0.8 = large).

**TABLE 2:** Semi‐quantitative angiogenesis scores (CD31 staining, 0–3 scale; mean ± SD; 95% CI) at baseline, Day 7, and Day 14 across groups.

Timepoint	Control (*n* = 10)	Exoline (*n* = 10)	Glow (*n* = 10)	Elysee (*n* = 10)	ANOVA *p*‐value	Post hoc comparisons (Cohen's *d*)
Day 0	0.50 ± 0.18 (95% CI: 0.38–0.62)	0.52 ± 0.20 (0.38–0.66)	0.54 ± 0.19 (0.41–0.67)	0.51 ± 0.18 (0.39–0.63)	0.79 (ns)	No significant differences
Day 7	0.72 ± 0.22 (0.56–0.88)	1.08 ± 0.28 (0.88–1.28)	0.72 ± 0.22 (0.56–0.88)	1.05 ± 0.27 (0.86–1.24)	< 0.001	Exoline > Elysee > Glow > Control (Exoline vs. Control *d* = −1.4)
Day 14	0.91 ± 0.29 (0.71–1.11)	2.41 ± 0.34 (2.16–2.66)	1.20 ± 0.30 (0.99–1.41)	1.64 ± 0.31 (1.42–1.86)	< 0.001	Exoline > Elysee > Glow > Control (Exoline vs. Control *d* = −4.8, very large)

*Note:* One‐way ANOVA with Tukey's post hoc test performed; Cohen's *d* values indicate effect sizes. Exoline exosome elicited strongest angiogenic response, followed by Elysee and Glow. Cohen's *d* represents standardized effect size (0.2 = small, 0.5 = medium, 0.8 = large).

## 4 Discussion

Extracellular vesicles (EVs) have recently attracted considerable attention in dermatology for their capacity to influence cutaneous repair and regeneration. By transferring bioactive molecules such as proteins, lipids, and microRNAs, EVs modulate processes central to skin healing, including fibroblast activation, keratinocyte migration, angiogenesis, and extracellular matrix remodeling. Evidence from preclinical and early clinical studies suggests that EV‐based interventions can accelerate wound closure, improve scar quality, and even support applications in aesthetic dermatology [30, 31, 32, 33, 34, 35, 36, 37]. Against this backdrop, our study aimed to evaluate the regenerative potential of three plant‐derived exosome formulations in a standardized rabbit wound model.

This study investigated the regenerative potential of three different plant‐derived exosome formulations: Exoline, Glow, and Elysee on the healing of laser‐induced full‐thickness cutaneous wounds in a standardized rabbit ear model. The healing process was assessed histologically using H&E and Masson Trichrome. Although all wounds, including untreated controls, achieved complete clinical closure by day 14, significant histological differences were observed between groups, highlighting the role of exosomes in enhancing the quality of repair rather than merely accelerating wound closure.

The control group demonstrated complete re‐epithelialization by day 14; however, collagen fibers remained sparse and loosely arranged, and CD31 expression was minimal, indicating limited neovascularization. This confirms that while physiological wound contraction leads to closure, the structural and vascular integrity of regenerated tissue is inferior in the absence of therapeutic intervention.

Among the treated groups, Exoline demonstrated an early advantage in angiogenesis at day 7, suggesting that its bioactive components may preferentially activate endothelial cells and initiate neovascularization during the proliferative phase of healing. Early collagen deposition was moderate, lower than Glow. By day 14, Glow surpassed Exoline in collagen maturation, exhibiting the most advanced collagen fibers with thick, parallel, and well‐organized bundles, indicating that Glow may contain molecular cargos more capable of sustaining fibroplasia and matrix remodeling through the remodeling phase.

In terms of angiogenesis, Exoline remained the most effective at both day 7 and day 14, followed by Elysee and Glow, consistent with robust early and sustained neovascularization. This late‐phase vascular activity highlights the ability of Exoline to support functional vascular integration and tissue remodeling, while Elysee provided intermediate angiogenic effects and Glow maintained moderate angiogenesis throughout.

Taken together, the data suggest that the three exosome formulations exert phase‐specific advantages in wound healing: Exoline facilitates early and sustained angiogenesis, Glow supports both early collagen deposition and late collagen maturation, and Elysee provides moderate angiogenic support during both early and late phases. These differences may be attributed to variations in their molecular cargo, including growth factors, lipids, and microRNAs, although the proprietary nature of commercial formulations limits precise mechanistic attribution. A further limitation is the lack of direct characterization data for the commercial exosome formulations used in this study. Information on particle size distribution, protein concentration, or RNA cargo was not available from the manufacturer, which restricts the ability to link observed biological effects to specific molecular properties. While the products are standardized by the suppliers to ensure consistency across batches, independent validation through nanoparticle tracking analysis, proteomic profiling, and RNA sequencing would strengthen future studies. Until such data are available, the interpretation of mechanistic differences between formulations must remain cautious. Nevertheless, the findings demonstrate that plant‐derived exosomes can influence both fibroblast‐driven collagen deposition and endothelial cell‐driven angiogenesis in a temporally distinct manner.

The clinical relevance of these findings lies in the potential to tailor exosome therapy to specific phases of wound healing. For instance, formulations like Glow may be most effective where robust collagen maturation is desired, whereas Exoline may be preferable for maximal angiogenic support throughout healing. Such a phase‐specific approach could enhance outcomes in chronic wounds, burns, or post‐surgical incisions where impaired angiogenesis or collagen deposition frequently compromises healing.

From a translational perspective, while the rabbit ear model provides valuable insights into dermal repair, the relatively short observation window of 14 days limits conclusions about scar maturation and long‐term tissue remodeling. Human wound healing often extends over weeks to months, particularly in chronic wounds or burns, and thus the present findings should be considered preliminary. Nonetheless, the demonstrated phase‐specific effects of exosomes suggest potential applications in diverse clinical scenarios, ranging from chronic ulcer management to cosmetic dermatology and reconstructive surgery. Clinical trials with extended follow‐up will be essential to determine whether these early histological benefits translate into durable functional improvements in humans.

Despite its strengths, this study has limitations. The observation period was limited to 14 days, preventing assessment of long‐term outcomes such as scar maturation, dermal tensile strength, and functional recovery of the repaired tissue. The analysis was also limited to histological and immunohistochemical endpoints; molecular assays such as VEGF, TGF‐β, or collagen type I gene expression would have provided a more mechanistic understanding of the differences between formulations. Furthermore, as with all animal models, extrapolation to humans must be made with caution given interspecies variations in immune response and dermal biology. The statistical analysis was strengthened by inclusion of effect sizes and 95% confidence intervals, which provide insight into the magnitude and precision of treatment effects beyond *p*‐values. These measures enhance the robustness of the findings and address concerns regarding reproducibility.

Future studies should incorporate longer follow‐up intervals to capture the remodeling and scar maturation phases, as well as molecular and proteomic profiling to identify the key bioactive components responsible for observed effects. Dose–response studies would also be valuable to determine the optimal concentration and frequency of exosome application. Comparative trials between plant‐derived exosomes and animal‐derived or synthetic extracellular vesicles could further clarify the translational advantages of each. Ultimately, standardization of commercial exosome formulations including details of plant source, isolation methods, and quantification of molecular cargo will be critical for reproducibility and regulatory approval. It should also be emphasized that the current discussion of potential molecular mechanisms remains speculative, as no direct evidence was obtained regarding the protein, lipid, or RNA cargo of the tested formulations. This represents an important limitation, and future research incorporating molecular or proteomic profiling will be required to substantiate these hypotheses.

## 5 Conclusion

This study demonstrates that plant‐derived exosomes significantly enhance cutaneous wound healing by promoting collagen deposition and angiogenesis in a time‐ and formulation‐specific manner. Exoline primarily accelerated both early and late angiogenesis, Glow was most effective for early collagen deposition and advanced late‐stage collagen maturation, and Elysee provided intermediate angiogenic effects. While all groups achieved complete clinical wound closure, exosome‐treated wounds exhibited superior histological organization, collagen architecture, and vascular density compared to controls. These findings highlight the therapeutic potential of plant‐derived exosomes as safe, cell‐free biologics for regenerative medicine and underscore the need for further molecular investigations and long‐term clinical studies to confirm efficacy and optimize treatment strategies. However, these results remain preliminary and are based on a preclinical rabbit model; validation through extended and human clinical trials is essential before clinical translation.

## Author Contributions

Dr. Noury Adel was the principal contributor to this work, responsible for the conceptualization, study design, Surgical procedure and injections, methodological framework, supervision, and major interpretation of results. The other authors supported data collection, analysis, and manuscript revisions. All authors have reviewed and approved the final manuscript.

## Funding

The authors have nothing to report.

## Ethics Statement

Experimental protocols followed the Guidelines for the Care and Use of Laboratory Animals and were approved by An Institutional Ethics Committee.

## Conflicts of Interest

The authors declare no conflicts of interest.

## Data Availability

The data that support the findings of this study are available from the corresponding author upon reasonable request.
